# Anti-Inflammatory and Anti-Diabetic Effect of Black Soybean Anthocyanins: Data from a Dual Cooperative Cellular System

**DOI:** 10.3390/molecules26113363

**Published:** 2021-06-02

**Authors:** Jin-Nam Kim, Sung Nim Han, Hye-Kyeong Kim

**Affiliations:** 1 Department of Food Science & Nutrition, The Catholic University of Korea, 43 Jibong-ro, Wonmi-gu, Bucheon 14662, Korea; hankne@nate.com; 2 Department of Food and Nutrition, Seoul National University, 1Gwanak-ro, Gwanak-gu, Seoul 08826, Korea; snhan@snu.ac.kr

**Keywords:** adipocyte, macrophage, inflammation, insulin resistance

## Abstract

Obesity is characterized by elevated infiltration of macrophages into adipose tissue, leading to the development of insulin resistance. The black soybean seed coat is a rich source of anthocyanins with antioxidative and anti-inflammatory activities. This study investigated the effects of black soybean anthocyanin extract (BSAn) on obesity-induced oxidative stress, the inflammatory response, and insulin resistance in a coculture system of hypertrophied 3T3-L1 adipocytes and RAW264 macrophages. Coculture of adipocytes with macrophages increased the production of reactive oxygen species and inflammatory mediators and cytokines (NO, MCP-1, PGE_2_, TNFα, and IL-6) and the release of free fatty acids but reduced anti-inflammatory adiponectin secretion. BSAn treatment (12.5, 25, 50, and 100 μg/mL) alleviated the coculture-induced changes (*p* < 0.001) and inhibited coculture-induced activation of JNK and ERK signaling (*p* < 0.01). BSAn also blocked the migration of RAW264.7 macrophages toward 3T3-L1 adipocytes. In addition, treatment with BSAn increased PPARγ expression and glucose uptake in response to insulin in hypertrophied 3T3-L1 adipocyte and RAW264.7 macrophage coculture (*p* < 0.01). These results demonstrate that BSAn attenuates inflammatory responses and improves adipocyte metabolic function in the coculture of hypertrophied 3T3-L1 adipocytes and RAW264.7 macrophages, suggesting the effectiveness of BSAn for obesity-induced insulin resistance.

## 1. Introduction

Obesity is a major public health problem because of its close association with insulin resistance, leading to several metabolic diseases [1]. Insulin resistance, an impaired response of the peripheral tissues to insulin, results in hyperglycemia, which is related to the development of type 2 diabetes (T2DM) and its complications, such as cardiovascular disease, retinopathy, and kidney failure [2]. Obesity is considered a chronic low-grade inflammatory state, suggesting that inflammation is a potential mechanism by which obesity leads to insulin resistance [3,4]. Several factors, such as hypoxia, oxidative stress, lipotoxicity, and endoplasmic reticulum stress, have been shown to contribute to the initiation of obesity-associated inflammation [5].

In obese adipose tissue, adipocytes undergo hypertrophy, leading to increased release of free fatty acids (FFA) and secretion of inflammatory cytokines, such as IL-6 and monocyte chemoattractant protein-1 (MCP-1) [6]. In addition, the infiltration of macrophages is increased, and adipose tissue macrophages display phenotype changes from M2 (alternatively activated macrophages) to M1 (classically activated macrophages), which secrete high levels of reactive oxygen species (ROS) and pro-inflammatory cytokines, including tumor necrosis factor-alpha (TNFα), interleukin (IL)-6, and IL-1β [7]. Adipocyte-derived FFA and MCP-1 and macrophage-derived TNFα have been suggested to establish a vicious cycle, enhancing inflammatory responses and insulin resistance in obese adipose tissue [8]. These interactions are closely associated with the activation of nuclear factor-κB (NF-κB) and mitogen-activated protein kinase (MAPK) pathways, including extracellular signal-regulated kinase (ERK) and c-Jun N-terminal kinase (JNK) [9,10]. Therefore, disrupting these interactions between adipocytes and macrophages is expected to reduce the inflammatory response in obesity, which could help prevent obesity-induced insulin resistance. Several types of drugs are currently being used in the clinic to treat T2DM. Thiazolidinediones, known as peroxisome proliferator-activated receptor gamma (PPARγ) agonists, enhance insulin sensitivity by blocking the actions of TNFα and increasing adiponectin secretion [11]. However, these agents have adverse effects, such as weight gain, fluid retention, and increased risk of heart failure and bone fracture [12]. With this in mind, much interest has been directed toward dietary phytochemicals with the potential to increase insulin sensitivity. Dietary phytochemicals are consumed regularly as part of the human diet, posing a low risk of adverse effects.

Black soybean (*Glycine max*) has traditionally been consumed as a health-promoting food and medicinal material in Asia. The black coat of this soybean has a high content of anthocyanins, in which cyanidin-3-glucoside is the major anthocyanin [13]. Previous studies reported the antioxidative [14], anti-inflammatory [15,16], and anti-obesity [17] effects of black soybean anthocyanin extract (BSAn). Black soybean seed coat extract also ameliorated hyperglycemia and insulin sensitivity in diabetic mice [18]. In addition, dietary cyanidin-3-glucoside decreased the serum concentration and gene expression of inflammatory cytokines in high-fat diet-fed and *db*/*db* mice [19]. However, the effects of BSAn on the interaction between adipocytes and macrophages in obese adipose tissue are not fully understood. In this study, we hypothesized that BSAn exhibits a beneficial effect for the treatment of obesity-induced insulin resistance by reducing inflammation and ameliorating adipocyte dysfunction in obese adipose tissue. To examine this hypothesis, we used a coculture system of hypertrophied 3T3-L1 adipocytes and RAW264.7 macrophages to model inflamed obese adipose tissue, in which macrophages infiltrate into hypertrophied adipocytes, and investigated the effects of BSAn treatment on oxidative stress, inflammatory changes, and adipocyte metabolic function associated with the insulin response.

## 2. Results

### 2.1. Black Soybean Anthocyanins Reduce the Production of Reactive Oxygen Species (ROS) in the Coculture of Adipocytes and Macrophages

**Figure 1:**
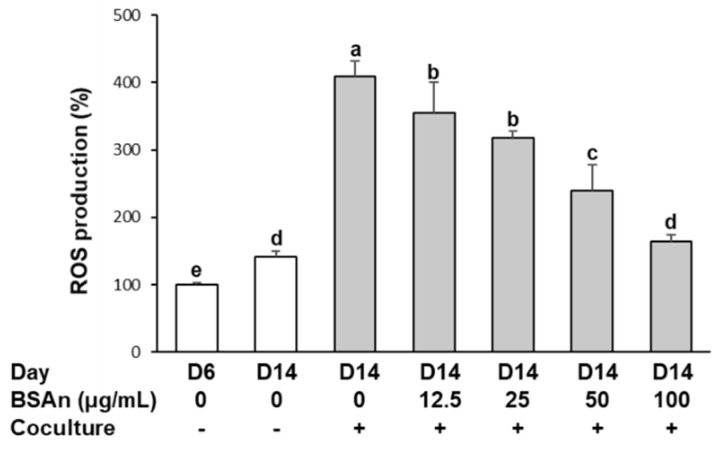
Effect of black soybean anthocyanin extract (BSAn) on reactive oxygen species (ROS) production in the coculture of adipocytes and macrophages. Hypertrophied 3T3-L1 adipocytes were cocultured with RAW264.7 macrophages for 24 h and then treated with BSAn for 24 h in the contact system. The level of ROS was determined by labeling with DCFH-DA, and the fluorescence intensity was analyzed by flow cytometry. Data are presented as mean ± SD of three independent experiments. ^a,b,c,d,e^ Means without the same letter are significantly different by ANOVA, followed by Duncan’s test (*p* < 0.001). +: adipocytes were cocultured with macrophages, -: adipocytes were cultured alone.

The effect of BSAn on the production of ROS is shown in Figure 1. ROS production was increased in hypertrophied 3T3-L1 adipocytes (D14) compared to mature adipocytes (D6). Moreover, coculture of hypertrophied adipocytes with RAW264.7 macrophages further increased ROS production to approximately 4-fold the level of the mature adipocyte. However, the enhanced production of ROS was significantly suppressed by BSAn treatment (*p* < 0.001). The ROS level was decreased by 40% and 60% with 50 and 100 μg/mL BSAn, respectively.

### 2.2. Black Soybean Anthocyanins Inhibit Transmigration of Macrophages to Adipocytes

**Figure 2:**
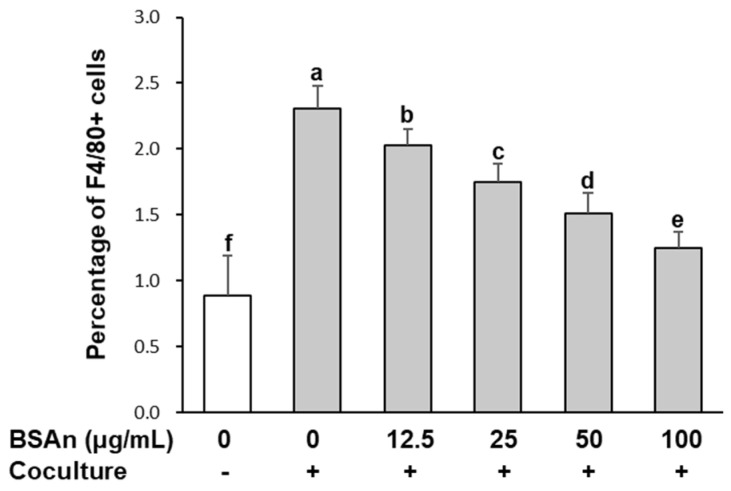
Effect of black soybean anthocyanin extract (BSAn) on transmigration of macrophages toward adipocytes. Hypertrophied 3T3-L1 adipocytes were cocultured with RAW264.7 macrophages for 24 h and then treated with BSAn for 24 h in the Transwell system. The number of cells labeled with F4/80 monoclonal antibody in a lower well was counted and result was expressed as percentage of F4/80+ cells. Data are presented as mean ± SD of three independent experiments. ^a,b,c,d,e,f^ Means without the same letter are significantly different by ANOVA, followed by Duncan’s test (*p* < 0.001). +: adipocytes were cocultured with macrophages, -: adipocytes were cultured alone.

The effect of BSAn on the transmigration of macrophages to 3T3-L1 adipocytes is shown in Figure 2. When RAW264.7 macrophages were cocultured with hypertrophied 3T3-L1 adipocytes, a marked increase in macrophage migration toward 3T3-L1 cells was observed. However, treatment with BSAn reduced the migratory ability of RAW264.7 macrophages in a dose-dependent manner (*p* < 0.001). While the percentage of RAW264.7 macrophages in the lower well of the control coculture was 2.3%, the percentage of F4/80 positive cells decreased to 1.75%, 1.51%, and 1.25% by 25, 50, and 100 μg/mL BSAn treatment, respectively.

### 2.3. Black Soybean Anthocyanins Alleviate Inflammatory Responses in the Coculture of Adipocytes and Macrophages

**Figure 3:**
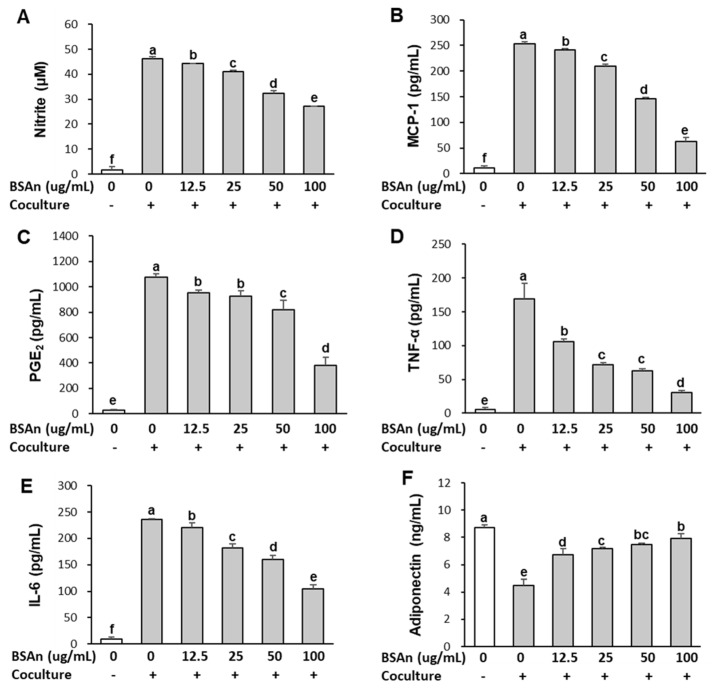
Effect of black soybean anthocyanin extract (BSAn) on the inflammatory response in the coculture of adipocytes and macrophages. Hypertrophied 3T3-L1 adipocytes were cocultured with RAW264.7 macrophages for 24 h and then treated with BSAn for 24 h in the contact system. The concentration of NO (**A**), MCP-1 (**B**), PGE_2_ (**C**), TNFα (**D**), IL-6 (**E**), and adiponectin (**F**) were measured in the coculture media by ELISA. Data are presented as mean ± SD of three independent experiments. ^a,b,c,d,e,f^ Means without the same letter are significantly different by ANOVA, followed by Duncan’s test (*p* < 0.001). +: adipocytes were cocultured with macrophages, -: adipocytes and macrophages were separately cultured and mixed.

**Figure 4:**
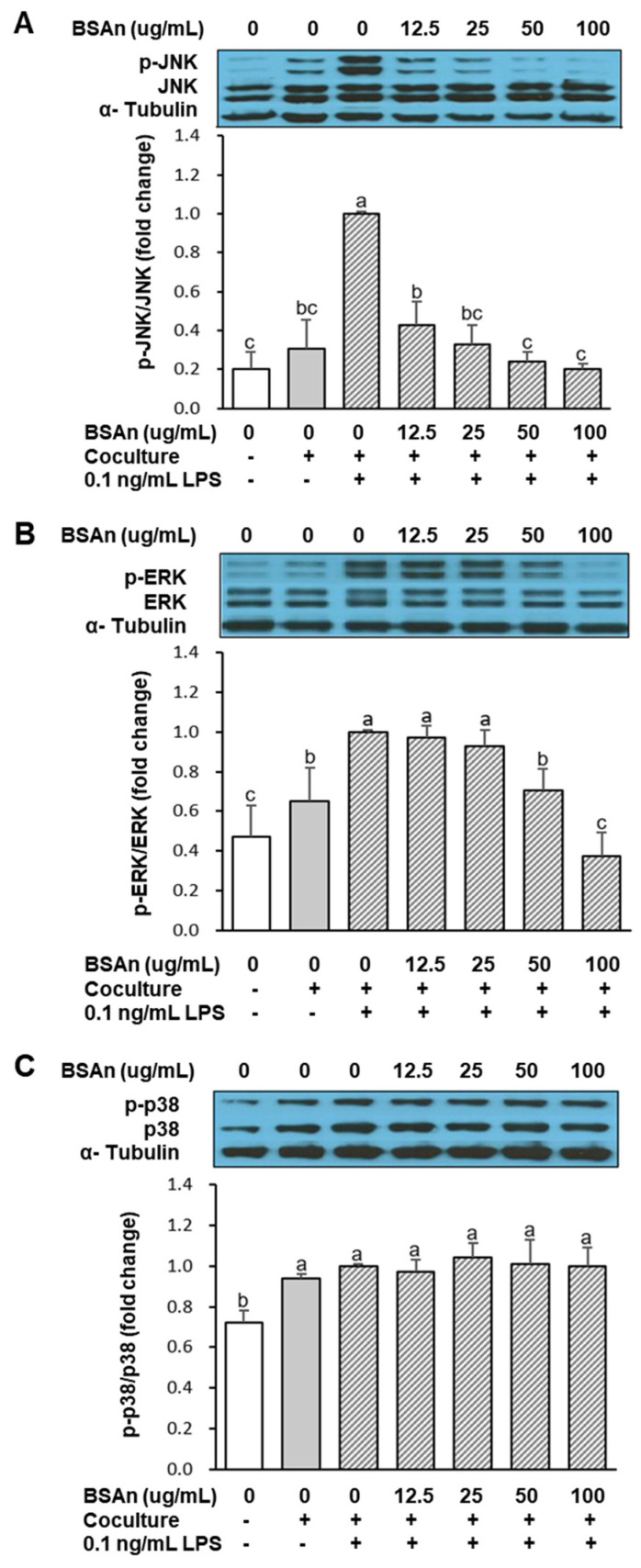
Effect of black soybean anthocyanin extract (BSAn) on the activation of MAPK in the coculture of adipocytes and macrophages. Hypertrophied 3T3-L1 adipocytes were cocultured with RAW264.7 macrophages for 24 h in the contact system. Cells were treated with BSAn for 24 h and later stimulated with LPS (0.1 μg/mL) for 15 min. The protein levels of JNK (**A**), ERK (**B**), and p38 (**C**) were measured by Western blot analysis. Data are presented as mean ± SD of three independent experiments. ^a,b,c^ Means without the same letter are significantly different by ANOVA, followed by Duncan’s test (*p* < 0.01). +: adipocytes were cocultured with macrophages (Coculture +) or cells were stimulated with LPS (0.1 ng/mL LPS +), -: adipocytes and macrophages were separately cultured and mixed (Coculture -) or cells were not stimulated with LPS (0.1 ng/mL LPS -).

Figure 3 shows the effect of BSAn on the secretion of nitric oxide (NO), MCP-1, prostaglandin E_2_ (PGE_2_), TNFα, IL-6, and adiponectin. The secretion of NO, MCP-1, PGE_2_, TNFα, and IL-6 from adipocytes and macrophages cultured separately was very low. Coculture of hypertrophied 3T3-L1 adipocytes and RAW264.7 macrophage resulted in a marked increase in these levels. However, treatment with BSAn inhibited the coculture-induced increase in NO, MCP-1, PGE_2_, TNFα, and IL-6, even at the lowest concentration (12.5 μg/mL) (*p* < 0.001). Meanwhile, the secretion of adiponectin was the highest in the control culture, and it was lowered by approximately 50% in the coculture of adipocytes and macrophages. BSAn treatment significantly increased the production of adiponectin dose-dependently. The adiponectin levels were increased by 47%, 58%, 63%, and 73% after treatment with 12.5, 25, 50, and 100 μg/mL BSAn, respectively, compared with the cocultured cells. These results indicate that BSAn modulates coculture-induced inflammatory responses, reducing inflammation. To investigate the signaling pathway responsible for modulation of the coculture-induced inflammatory response by BSAn, the effect of BSAn on MAPK activation was examined. As shown in Figure 4, coculture of hypertrophied adipocytes with RAW264.7 macrophages increased the phosphorylation of MAPK and lipopolysaccharide (LPS) treatment stimulated the increase in phosphorylated ERK and JNK (*p* < 0.01). However, pretreatment with BSAn inhibited the LPS-induced phosphorylation of these two MAPK in cocultured cells, presenting a stronger inhibitory effect on JNK than ERK. On the contrary, the phosphorylation of p38 was not affected by either LPS stimulation or BSAn treatment in cocultured cells.

### 2.4. Black Soybean Anthocyanins Suppress Lipolysis in Adipocytes Cocultured with Macrophages

**Figure 5:**
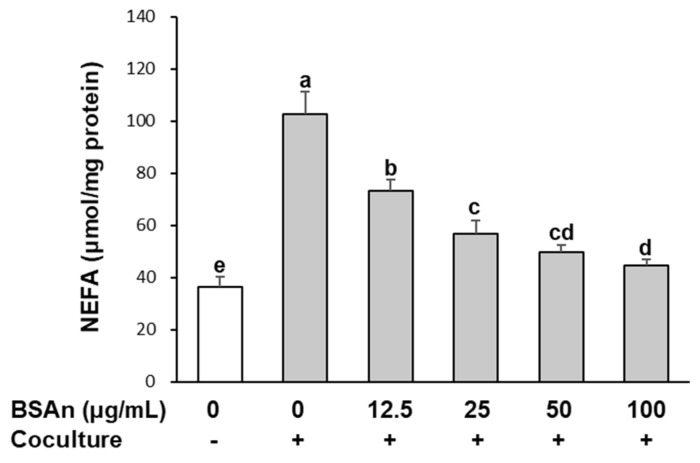
Effect of black soybean anthocyanin extract (BSAn) on the release of free fatty acids in the coculture of adipocytes and macrophages. Hypertrophied 3T3-L1 adipocytes were cocultured with RAW264.7 macrophages for 24 h and then treated with BSAn for 24 h in the contact system. Concentration of non-esterified fatty acid (NEFA) in the coculture medium was measured by NEFA kits. Data are presented as mean ± SD of three independent experiments. ^a,b,c,d,e^ Means without the same letter are significantly different by ANOVA, followed by Duncan’s test (*p* < 0.001). +: adipocytes were cocultured with macrophages, -: adipocytes and macrophages were separately cultured and mixed.

To investigate the effect of BSAn on the FFA efflux that is closely associated with insulin resistance, the release of non-esterified fatty acids (NEFA) into the culture medium was measured in adipocytes cocultured with macrophages. As shown in Figure 5, coculture of hypertrophied 3T3-L1 adipocytes with RAW264.7 macrophages increased the NEFA release by 3-fold compared with the control culture. However, this increase was significantly reduced by BSAn treatment in a dose-dependent manner (*p* < 0.001). The NEFA level was decreased by 56% with 100 μg/mL BSAn relative to the control coculture.

### 2.5. Black Soybean Anthocyanins Stimulate Glucose Uptake in Insulin-Resistant Adipocytes

**Figure 6:**
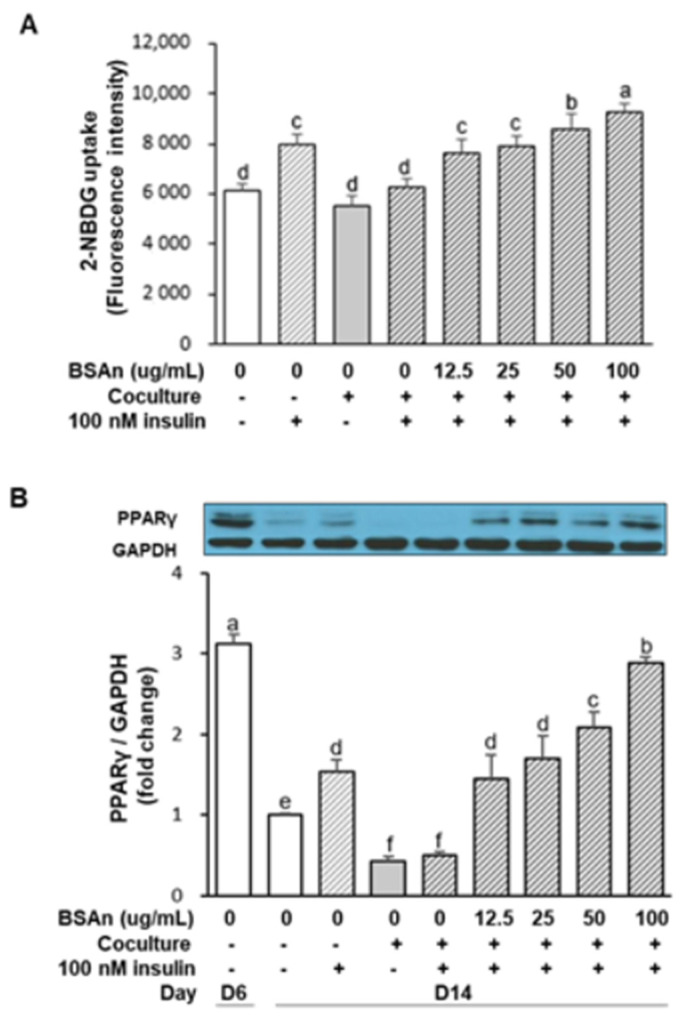
Effect of black soybean anthocyanin extract (BSAn) on the glucose uptake (**A**) and PPARγ expression (**B**) in the coculture of adipocytes and macrophages. Hypertrophied 3T3-L1 adipocytes were cocultured with RAW264.7 macrophages for 24 h in the contact system. Cells were treated with BSAn for 24 h, and insulin (100 nM) was added 30 min before the end of the treatment. For glucose uptake assay, cells were incubated with the glucose analog, 2-NBDG (20 μM), for 30 min and the fluorescence was measured. PPARγ expression was measured by Western blot analysis. Data are presented as mean ± SD of three independent experiments. ^a,b,c,d,e,f^ Means without the same letter are significantly different by ANOVA, followed by Duncan’s test (*p* < 0.01). +: adipocytes were cocultured with macrophages (Coculture +) or cells were treated with insulin (100 nM insulin +), -: adipocytes and macrophages were separately cultured and mixed (Coculture -) or cells were not treated with insulin (100 nM insulin -).

The effect of BSAn on glucose uptake in the coculture of adipocytes and macrophages is shown in Figure 6A. The incorporation of 2-[*N*-(7-nitrobenz-2-oxa-1,3-diazol-4-yl)-amino]-2-deoxy-d-glucose (2-NBDG) in cocultured cells was not affected in response to insulin, whereas 2-NBDG uptake was increased by insulin when the cells were cultured separately. This result indicates that the coculture of hypertrophied 3T3-L1 adipocytes and RAW264.7 macrophages induced insulin resistance. However, treatment with BSAn increased the 2-NBDG uptake by 35% and 45% with 50 and 100 μg/mL BSAn, respectively, compared to the control coculture (*p* < 0.01). To identify a possible mechanism for the increase in glucose uptake observed in BSAn-treated adipocytes, we investigated whether the expression of PPARγ, a major transcription factor regulating the expression of genes associated with mature adipocyte function, was involved. As shown in Figure 6B, the expression of PPARγ was maximal in mature adipocytes (D6) and decreased to 30% in hypertrophied adipocytes, which was consistent with a previous report [20]. The PPARγ expression was decreased further when hypertrophied adipocytes were cocultured with macrophages. Insulin enhanced the expression of PPARγ in hypertrophied adipocytes, but it could not increase PPARγ expression in cocultured cells. However, BSAn treatment of cocultured cells restored PPARγ expression at the concentration of 12.5 μg/mL BSAn. PPARγ expression increased, reaching 67% and 91% of the mature adipocyte level following treatment with 50 and 100 μg/mL BSAn, respectively.

## 3. Discussion

In the present study, we investigated the effects of BSAn on inflammatory responses and adipocyte function in hypertrophied adipocytes cocultured with macrophages to model obese adipose tissue. The results demonstrate that BSAn has multiple effects on inflammatory responses, macrophage infiltration, lipolysis, and insulin-stimulated glucose uptake with regards to insulin sensitivity.

Fat accumulation in adipose tissue can increase oxidative stress, which leads to obesity-associated complications by causing dysregulated production of inflammatory mediators and cytokines [21]. Oxidative stress is caused by an imbalance between ROS production and the antioxidant defense system. This study shows that ROS production was higher in hypertrophied adipocytes than mature adipocytes and was further increased by the coculture with macrophages. ROS produced by hypertrophied adipocytes seemed to be acting as signaling molecules and contribute to the activation of macrophages, exacerbating the generation of ROS in macrophages [22]. In our results, BSAn treatment effectively inhibited coculture-induced ROS production. Anthocyanins are potential scavengers of ROS. Cyanidin-3-glucoside, which comprised 68.3% of the BSAn used in this study, was reported to have the strongest radical quenching activity among anthocyanins [23]. In addition, black soybean anthocyanin has been shown to enhance the activities of antioxidant defense enzymes in high-fat-induced obese mice [24]. Thus, suppression of ROS by BSAn treatment might be attributed to the upregulation of cellular defense enzymes and scavenging of ROS.

Coculture of hypertrophied 3T3-L1 adipocytes and RAW264.7 macrophages has been used as ae model of adipose tissue inflammation in which pro-inflammatory cytokines are upregulated [20]. In the present study, BSAn treatment suppressed macrophage migration and inhibited the coculture-induced production of inflammatory mediators (NO, PGE_2_) and inflammatory cytokines, including TNFα, IL-6, and MCP-1. Considering that infiltrated macrophages are an important source of pro-inflammatory mediators in adipose tissue, suppression of migration by BSAn could ameliorate obesity-related inflammation. It has been reported that adipocyte-derived MCP-1 enhances macrophage infiltration into adipose tissue and increases TNFα expression [25]. Our data indicate that BSAn inhibits macrophage migration by suppressing MCP-1 production, thereby decreasing inflammatory mediators and cytokines.

Toll-like receptor 4 (TLR-4), widely known as the receptor for LPS, is involved in the main pathway for stimulating inflammatory responses. It is expressed in various cells, including macrophages and adipocytes [9]. Once stimulated, TLR-4 activates intracellular NF-κB and MAPK signaling, leading to upregulation of inflammatory cytokine production [26]. We previously reported that BSAn suppressed production of NO, PGE_2_, TNFα, and IL-6 in LPS-stimulated RAW264.7 macrophages by inhibiting activation of MAPKs [27]. Suppression of NF-κB signaling by cyanidin-3-glucoside, the main constituent of BSAn, was proposed to reduce palmitate-induced inflammation in hypertrophied 3T3-L1 cells [28]. In this study, stimulation with LPS or coculture increased the phosphorylation of all MAPKs, indicating TLR-4 activation. BSAn treatment suppressed JNK and ERK activation but did not affect p38 in the coculture of adipocytes and macrophages. Thus, inhibition of JNK and ERK signaling seemed to be a major mechanism for the suppression of obesity-induced inflammation by BSAn. Considering that JNK and ERK function as central mediators of inflammation-evoked insulin resistance in adipose tissue [29], inhibition of JNK and ERK signaling by BSAn could be associated with the effectiveness of BSAn for ameliorating obesity-induced insulin resistance.

Macrophage-derived TNFα increases pro-inflammatory cytokine production and stimulates lipolysis in adipocytes [30]. Contrariwise, adipocyte-derived FFA promotes the release of inflammatory factors by activating TLR-4 signaling in macrophages [31]. In addition, it was reported that hypertrophied adipocytes treated with FFA secrete chemotactic signals, in particular MCP-1, that induce macrophage migration [32]. These interactions between macrophages and adipocytes have been suggested to establish a vicious cycle, enhancing inflammatory responses and insulin resistance in obese adipose tissue [8]. The suppressive effect of BSAn on NEFA release as well as TNFα and MCP-1 production, observed in this study, indicates that BSAn could be effective in alleviating obesity-related inflammation by reducing the cross-talk between macrophages and adipocytes.

Adipose tissue plays an important role in regulating whole-body insulin sensitivity through the secretion of FFA and adipokines [33]. Hypertrophied adipocytes increase basal lipolysis in obesity, resulting in the elevation of FFA release, which has been suggested to cause insulin resistance by modulating adipokine secretion and inducing ectopic lipid accumulation in the liver and skeletal muscle (lipotoxicity) [34]. In addition, FFA inhibited insulin-stimulated glucose uptake and insulin signaling [35]. In this study, NEFA release was further increased in the coculture of hypertrophied adipocytes and macrophages, which can be attributed to the enhanced lipolysis by macrophage-derived TNFα, as described above. On the contrary, adiponectin release from adipocytes was decreased by coculture with macrophages. Adiponectin exerts an anti-inflammatory effect by suppressing the synthesis of inflammatory cytokines and promoting macrophage polarization toward the anti-inflammatory M2 phenotype [36]. It has been reported that the circulating level of adiponectin is lowered in obesity, and adiponectin treatment decreases hyperglycemia and improves insulin sensitivity by acting on skeletal muscle and the liver [36]. Our results show that BSAn treatment restored the coculture-induced suppression of adiponectin production and inhibition of NEFA release. These effects can contribute to the antidiabetic effect of BSAn by ameliorating hyperglycemia and improving insulin sensitivity.

Glucose uptake into peripheral tissues is the most effective way to control blood glucose levels. Glucose uptake is low under the basal condition, but insulin secretion stimulates glucose uptake into the target tissues. To evaluate the efficacy of BSAn for the treatment of T2DM, we examined the effects of BSAn on 2-NBDG uptake in hypertrophied adipocytes that were cocultured with macrophages. Unlike separately cultured adipocytes, adipocytes cocultured with macrophages failed to increase glucose uptake in response to insulin, which indicated the induction of insulin resistance. Our result shows that BSAn could completely counteract the impaired glucose uptake in response to insulin in the coculture system. To elucidate the mechanism for the improved insulin sensitivity of adipocytes observed with BSAn treatment, we investigated the changes in PPARγ expression. PPARγ is highly expressed in white adipose tissue and regulates adipocyte differentiation, fatty acid storage, and glucose metabolism [37]. PPARγ activation improves insulin resistance by regulating the expression of adipokines and genes encoding proteins involved in glucose and lipid metabolism [38]. Thiazolidinediones are PPARγ agonists that have been used for the treatment of T2DM. Anthocyanins have been shown to activate PPARγ by functioning as agonistic ligands [39]. In this study, BSAn increased the expression of PPARγ that was suppressed by coculture with macrophages. Considering that insulin induces PPARγ expression in adipocytes [40], this result suggests that BSAn treatment in cocultured cells counteracts the impaired response to insulin, thereby increasing glucose uptake. In addition, increased adiponectin levels in BSAn-treated adipocytes could be attributed to the upregulation of PPARγ gene expression, considering that adiponectin has a peroxisome proliferator response element in its promoter [41]. A limitation of our study is that the effect on the insulin signaling pathway and GLUT4 expression with regard to glucose transport was not investigated. Nevertheless, to our best knowledge, this is the first study to show the direct effects of BSAn on inflammatory responses and adipocyte metabolic function during the interactions of adipocytes and macrophages.

In summary, BSAn treatment suppressed macrophage transmigration and the production of ROS and pro-inflammatory mediators, whereas adiponectin production increased in hypertrophied adipocytes cocultured with macrophages, which might be mediated via inhibiting JNK and ERK1/2 signaling. Furthermore, BSAn could decrease FFA release and recover impairment of insulin-stimulated glucose uptake in cocultured cells by increasing PPARγ expression. Therefore, BSAn has the potential to be used as a therapeutic agent to ameliorate obesity-associated insulin resistance by alleviating inflammatory changes and adipocyte dysfunction.

## 4. Materials and Methods

### 4.1. Chemicals and Preparation of BSAn

Dulbecco’s modified Eagle medium (DMEM), fetal bovine serum (FBS), bovine calf serum, penicillin-streptomycin, RPMI 1640 medium, and 2-NBDG were purchased from Invitrogen (Carlsbad, CA, USA). The enzyme immunoassay (EIA) kits for PGE_2_, TNFα, IL-6, MCP-1, and adiponectin were obtained from R&D Systems (Minneapolis, MN, USA). Fc blocking antibody was obtained from BD Biosciences (San Jose, CA, USA). Antibodies specific for PPARγ and PE/Cyanine5 anti-mouse F4/80 were purchased from Santa Cruz Biotechnology (Santa Cruz, CA, USA) and eBioscience (San Diego, CA, USA), respectively. Other antibodies were purchased from Cell Signaling Technology (Beverly, MA, USA). LPS and all other chemicals were purchased from Sigma-Aldrich (St. Louis, MO, USA).

BSAn extract was provided by the Rural Development Administration (RDA), Republic of Korea. It was extracted from the seed coat of the black soybean cultivar Cheongja3. The procedures for extraction and identification of major compounds were described in a previous study [13]. Briefly, hand-peeled seed coats (500 g) were extracted with 80% ethanol (0.1% acetic acid) for 2 days, filtered through a 0.45 μm filter, and concentrated to obtain the crude extract. The crude extract was freeze-dried to remove residual solvent and to yield red powder (7.1 g). HPLC chromatogram analysis of the extract indicated three major anthocyanins (cyanidin-3-*O*-glucoside, delphinidin-3-*O*-glucoside, and petunidin-3-*O*-glucoside) represented about 90% of the total peak area and 6 minor anthocyanins detected were less than 5%, while isoflavones were not detected. The amount of cyanidin-3-*O*-glucoside, delphinidin-3-*O*-glucoside, and petunidin-3-*O*-glucoside in the extract were 582.9 mg, 214.7 mg, and 55.8 mg per 1 g extract. Therefore, BSAn extract contains more than 85.3% anthocyanins. The composition of anthocyanins was cyanidin-3-*O*-glucoside (68.3%), delphinidin-3-*O*-glucoside (25.2%), and petunidin-3-*O*-glucoside (6.5%), as determined from the peak-area ratios in the HPLC chromatogram.

### 4.2. Cell Culture

3T3-L1 mouse embryo fibroblasts and RAW264.7 macrophage cells were purchased from the American Type Culture Collection (ATCC, Manassas, VA, USA). Cell culture was performed as previously described [42]. Briefly, preadipocytes were grown in 10% bovine calf serum/DMEM until 2 days after confluence and differentiated with induction medium containing 10% FBS, 0.5 µM isobutylmethylxanthine, 1 µM dexamethasone, and 167 nM insulin (D0). After 2 days, cells were cultured with 10% FBS/DMEM and 167 nM insulin for another 2 days and then maintained with 10% FBS/DMEM. Cells displayed lipid-filled mature adipocyte phenotype approximately after D5, and lipid accumulation continued thereafter. Adipocytes from D14 to D20 were used as hypertrophied 3T3-L1 adipocytes. 3T3-L1 adipocytes and RAW264.7 macrophages were cocultured in two different ways, as previously described [9]. In the contact system, RAW264.7 macrophages (5 × 10^5^ cells/mL) were plated in culture dishes containing serum-starved and hypertrophied 3T3-L1 cells and then incubated in serum-free DMEM for 24 h. As a control culture, 3T3-L1 cells and RAW264.7 macrophages were cultured separately under the same conditions. Cocultured cells were treated with the indicated concentrations of BSAn or 0.1% dimethyl sulfoxide as a control. In the Transwell system, cells were cultured using Transwell inserts with a 0.4 μm porous membrane (Corning, NY, USA) to separate adipocytes and macrophages.

### 4.3. Measurement of Intracellular ROS

Intracellular ROS production was measured using the fluorescent probe 2,7-dichlorofluorescein diacetate (DCFH-DA). DCFH-DA is hydrolyzed to DCFH by deacetylase within the cells and oxidized by various intracellular ROS to DCF, a fluorescent compound. Hypertrophied 3T3-L1 cells and RAW264.7 macrophages were cocultured using the contact system and treated with BSAn (12.5, 25, 50, 100 μg/mL) for 24 h. The cells were washed with serum-free medium and treated with 20 μM DCFH-DA at 37 °C for 30 min in a CO_2_ incubator. The DCF level was measured using a flow cytometer (Cytomics FC500, Beckman Coulter, Brea, CA, USA).

### 4.4. Macrophage Migration Assay

F4/80 is a murine macrophage-surface glycoprotein and has been widely used to characterize macrophage populations in immunological studies [43]. The effect of BSAn on the transmigration of macrophage to 3T3-L1 adipocytes was investigated in the Transwell coculture system. RAW264.7 cells (5 × 10^4^ cells/well) were placed in the upper Transwell inserts of a culture chamber to separate 3T3-L1 cells in a lower well. After incubation with BSAn (12.5, 25, 50, 100 μg/mL) for 24 h, the cells in the lower well were harvested and incubated with Fc block (20 μg/mL) at 4 °C for 15 min to prevent non-specific Fc receptor binding. For macrophage assessment, cells were incubated with fluorochrome-conjugated F4/80 PE/Cyanine5 antibody for an additional 15 min and fixed with 1% paraformaldehyde. Cells were counted using a flow cytometer, and transmigration of macrophages was quantitated by counting the number of cells labeled with F4/80 antibody in every 10,000 cells and result was expressed as the percentage of F4/80+ cells.

### 4.5. Measurement of NO, PGE_2_, and Cytokine Production

Hypertrophied 3T3-L1 cells and RAW264.7 macrophages were cocultured by the contact systems, and the coculture was then treated with BSAn (12.5, 25, 50, 100 μg/mL). After 24 h incubation, the culture supernatants were collected and used for assays. The nitrite accumulation in each culture supernatant was measured by the Griess method. Briefly, 100 μL of supernatant was mixed with the same volume of Griess reagent (1% sulfanilamide in 5% phosphoric acid, 0.1% *N*-(1-naphthyl) ethylenediamine in H_2_O) and incubated at room temperature for 10 min. The absorbance was measured at 540 nm. The nitrite concentrations were calculated from a standard sodium nitrite curve. The concentrations of PGE_2_, MCP-1, TNFα, IL-6, and adiponectin in the cell culture supernatants were determined using EIA kits according to the respective manufacturer’s instructions.

### 4.6. Western Blotting of MAPK and PPARr

After hypertrophied 3T3-L1 cells and RAW264.7 macrophages were cocultured, cells were treated with BSAn (12.5, 25, 50, 100 μg/mL) for 24 h. For the assessment of MAPK activation, LPS (0.1 μg/mL) was added 15 min before the end of treatment. For the measurement of PPARγ expression, insulin (100 nM) was added 30 min before the end of incubation. The cells were collected and lysed in a cold lysis buffer, pH 7.4 (20 mM Tris-HCl, 150 mM NaCl, 1 mM Na_2_EDTA, 1 mM EGTA, 1% NP-40, 1% sodium deoxycholate, 2.5 mM sodium pyrophosphate, 1 mM β-glycerophosphate, 1 mM Na_3_VO_4_, and 1 μg/mL leupeptin) and kept on ice for 15 min. After centrifugation at 10,000× *g*, 4 °C, for 10 min, aliquots of lysates (30 μg protein/lane) were separated by 10% SDS-PAGE and transferred onto nitrocellulose membranes (Bio-Rad, Hercules, CA, USA). After blocking with 5% skim milk in PBS/0.1% Tween 20 for 1 h, the membrane was incubated overnight with specific primary antibodies (ERK, phospho-ERK, JNK, phospho-JNK, p38, phospho-p38, and PPARγ) at 4 °C and then with a secondary horseradish peroxidase-conjugated antibody at room temperature for 1 h. Immunoblots were developed with the ECL system (Ab Frontier, Seoul, Korea). Band intensities were measured with a FluorChem densitometer using the ImageJ software (National Institute of Health, Bethesda, MD, USA).

### 4.7. Lipolysis Assay

Lipolysis was measured by the NEFA released into the medium from adipocytes. Hypertrophied 3T3-L1 cells were cocultured with RAW264.7 macrophages for 24 h in the contact system. After washing with PBS, cells were incubated in Krebs−Ringer bicarbonate buffer (119 mM NaCl, 4.8 mM KCl, 1.28 mM CaCl_2_, 1.2 mM KH_2_PO_4_, 1.2 mM 7H_2_O·MgSO_4_, 0.25 mM NaHCO_3_, 5 mM glucose, 4% bovine serum albumin, pH 7.4) containing BSAn (12.5, 25, 50, 100 μg/mL) for 24 h. The concentration of NEFA in the medium was measured using a commercial kit (Wako, Osaka, Japan), and pellets were assayed for protein concentration for calibration.

### 4.8. Glucose Uptake Assay

RAW264.7 cells were placed onto the hypertrophied 3T3-L1 cells cultured in 96-well fluorescence plates. After 24 h incubation, cells were pre-incubated with serum-free DMEM and treated with BSAn (12.5, 25, 50, 100 μg/mL) for 24 h. Insulin (100 nM) was added 30 min before the end of incubation. After washing with serum-free DMEM, 20 µM of the fluorescent glucose analog, 2-NBDG, was added and incubated for 30 min. Cells were washed with cold PBS to remove free 2-NBDG. The fluorescence retained in the cell monolayers was measured using a fluorescence microplate reader, with an excitation wavelength of 465 nm and an emission wavelength of 540 nm.

### 4.9. Statistical Analysis

Statistical analysis was performed by one-way ANOVA followed by Duncan’s multiple range test at *p* < 0.05 using SAS software (Version 9.4; SAS Institute, Inc., Cary, NC, USA). The results are presented as mean ± standard deviation (SD). All experiments were performed at least three times.

## Data Availability

Data is contained within the article.

## Sample Availability

Samples of the compounds are not available from the authors.

## Abbreviations

2-NBDG	2-[*N*-(7-nitrobenz-2-oxa-1,3-diazol-4-yl)-amino]-2-deoxy-d-glucose
BSAn	black soybean anthocyanin extract
DCFH-DA	2,7-dichlorofluorescein diacetate
DMEM	Dulbecco’s modified Eagle medium
ERK	extracellular signal-regulated kinase
FBS	fetal bovine serum
FFA	free fatty acids
IL	interleukin
JNK	c-Jun N-terminal kinase
LPS	lipopolysaccharide
MAPK	mitogen-activated protein kinase
MCP-1	monocyte chemoattractant protein 1
NEFA	non-esterified fatty acids
NF-κB	nuclear factor-κB
NO	nitric oxide
PGE_2_	prostaglandin E_2_
PPARγ	peroxisome proliferator-activated receptor gamma
ROS	reactive oxygen species
T2DM	type 2 diabetes
TLR-4	Toll-like receptor 4
TNFα	tumor necrosis factor-alpha
